# Foreign Bodies in the Rectum

**Published:** 1844-11-02

**Authors:** 


### FOREIGN BODIES IN THE RECTUM.

M. Maisonneuve related to the "Societè Medico Pratique," the case of a man—a patient of M. Cloquet's—who had introduced a tumbler into his rectum. In order to extract it, M. Cloquet dilated the anus with six fingers, which being insufficient to dilate it to the required extent, MM. Maisonneuve and Huguier, who were present, each added four fingers. The fourteen fingers enlarged the anal surface to such a degree, as to allow the tumbler to be seen. The bottom of the tumbler was directed upwards, and the open part downwards. The man was then told to bear down, as if for defaction, and the glass was expelled. This case is a most remarkable exampleof the extent to which the anus may be dilated, without injury to the sphinters.

A few weeks previous, M. Cloquet had had under his care another individual, who had introduced a Flemish beer-glass (shaped like our champagne glasses) into his rectum. The glass was seized with forceps, but broke into many pieces. In order to get the lower part out, it was found necessary to turn it, as the open broken part was turned downwards. The man died in the course of a few days.

M. Thierry narrated a case which occurred to Dupuytren. A man had introduced a square preserve-pot into the rectum, the open part being superior. Dupuytren seized hold of the rim by means of a blunt hook covered with chamois leather, and thus extracted it.—*Ibid, from Gazette des Hôpitaux.*

